# *Guanine Nucleotide Exchange Factor 7B* (*RopGEF7B*) is involved in floral organ development in *Oryza sativa*

**DOI:** 10.1186/s12284-018-0235-0

**Published:** 2018-07-30

**Authors:** Jiaqing Huang, Huili Liu, Thomas Berberich, Yuting Liu, Li-zhen Tao, Taibo Liu

**Affiliations:** 1 State Key Laboratory for Conservation and Utilization of Subtropical Agro-bioresources, South China Agricultural University, Guangzhou, 510642 China; 2 Guangdong Provincial Key Laboratory of Protein Function and Regulation in Agricultural Organisms, College of Life Sciences, South China Agricultural University, Guangzhou, 510642 China; 3 Senckenberg Biodiversity and Climate Research Center, Georg-Voigt-Str. 14–16, D-60325 Frankfurt am Main, Germany

**Keywords:** Agronomic traits, Floral development, OsRAC, OsRopGEF, Rice

## Abstract

**Background:**

RAC/ROP GTPase are versatile signaling molecules controlling diverse biological processes including cell polarity establishment, cell growth, morphogenesis, hormone responses and many other cellular processes in plants. The activities of ROPs are positively regulated by guanine nucleotide exchange factors (GEFs). Evidence suggests that *RopGEFs* regulate polar auxin transport and polar growth in pollen tube in *Arabidopsis thaliana*. However, the biological functions of rice *RopGEFs* during plant development remain largely unknown.

**Results:**

We investigated a member of the *OsRopGEF* family, namely *OsRopGEF7B. OsRopGEF7B*_*pro*_*:GUS* analysis indicates that *OsRopGEF7B* is expressed in various tissues, especially in the floral meristem and floral organ primordia. Knock-out and -down of *OsRopGEF7B* by T-DNA insertion and RNA interference, respectively, predominantly caused an increase in the number of floral organs in the inner whorls (stamen and ovary), as well as abnormal paleae/lemmas and ectopic growth of lodicules, resulting in decline of rice seed setting. Bimolecular fluorescence complement (BiFC) assays as well as yeast two-hybrid assays indicate that OsRopGEF7B interacts with OsRACs.

**Conclusions:**

*OsRopGEF7B* plays roles in floral organ development in rice, affecting rice seed setting rate. Manipulation of *OsRopGEF7B* has potential for application in genetically modified crops.

**Electronic supplementary material:**

The online version of this article (10.1186/s12284-018-0235-0) contains supplementary material, which is available to authorized users.

## Background

RAC/ROPs, plant Rho-like small G proteins, are multi-functional signaling switches regulating many cellular processes in plants, affecting leaf epidermal cell morphogenesis, polarized cell growth in pollen tubes and root hairs, and hormone and defense-related responses (Yang and Fu 2007; Yalovsky et al. 2008; Wu et al. 2011; Nibau et al. 2013; Huang et al. 2014). RAC/ROPs are essential signaling molecules that switch between a GTP-bound active form and a GDP-bound inactive form (Bourne et al. 1990, 1991; Wu et al. 2011). Activation of GTPases depends on guanine nucleotide exchange factors (GEFs) that stimulate the exchange of GDP- to GTP-bound form (Cherfils and Chardin 1999; Shichrur and Yalovsky 2006; Wu et al. 2011). It was shown that RAC/ROPs utilize mostly a plant-specific family of GEFs named RopGEFs for activation in plant kingdom (Berken et al. 2005).

*Arabidopsis* contains 14 *RopGEF*s in its genome, sequentially termed *RopGEF1* to *RopGEF14*, sharing a conserved PRONE domain for GEF catalytic activity. Transient expression analyses provide evidence that *RopGEF1* and *RopGEF12* regulate polarized pollen tube growth (Gu et al. 2006; Zhang and McCormick 2007). ABA-mediated degradation of RopGEF1 also plays an important role in ABA-mediated inhibition of lateral root growth (Li et al. 2016). Most recently, our group reported that RopGEF1 is very important in the plant early development via affecting cell polarity and polar auxin transport (Liu et al. 2017). The study of a *ropgef1ropgef4* double mutant suggests that RopGEF1 and RopGEF4 are specific regulators of ROP11 function in ABA-mediated stomatal closure (Li and Liu 2012). *Arabidopsis* full-genome chip transcriptome assay results in combination with physiological studies further support that RopGEF10 negatively regulates ABA responses by inducing a particular subset of genes associated with stress responses (Xin et al. 2005). Previously, our group has reported that *Arabidopsis* RopGEF7, is specifically expressed in the quiescent center (QC) precursors during embryogenesis and in the QC of postembryonic roots, regulating PLT-mediated maintenance of root meristem by connecting RopGEF-regulated RAC/ROP signaling and polar auxin transport (Chen et al. 2011). Furthermore, ROP3 interacts directly with RopGEF7 (Chen et al. 2011) and regulates *Arabidopsis* embryo development and seedling growth via affecting the polar auxin transport and thus controlling the establishment of auxin maxima (Huang et al. 2014).

Rice contains 11 *RopGEFs* in its genome (Berken et al. 2005; Gu et al. 2006; Yoo et al. 2011). It was shown that PRONE-type RacGEFs in rice may play a role in the activation of OsRac1 in disease resistance response (Kawasaki et al. 2009). The expression levels of some OsRacGEFs were affected by treatment with sphingolipid elicitors (SE), implying that some OsRacGEFs may be regulated at the transcription level during plant response to stress (Kawasaki et al. 2009). OsRopGEF10 predominantly expressed in newly developed leaves before the appearance from the leaf sheath and regulated small papillae development (Yoo et al. 2011). However, studies on *RopGEFs* in rice are still limited.

In this work, we examined a *RopGEF* member in rice, namely *OsRopGEF7B,* which is a homolog of *AtRopGEF7*. *AtRopGEF7* was reported to be essential for root meristem maintenance (Chen et al. 2011). Our results indicated that *OsRopGEF7B* was highly expressed in root meristem, floral meristem, floral organ primordia, and the inner floral organs. We further explored the roles of *OsRopGEF7B* in rice development by analyzing the *osropgef7b-1* mutant and *OsRopGEF7B-RNAi* plants, both of which displayed variedly defective phenotypes, including increased number of stamens and ovaries, abnormal palea/lemma, and shortened plant height. Taken together, these results implicate that *OsRopGEF7B* plays a role in regulating floral organ development, thus subsequently affects rice seed setting rate.

## Results

### *OsRopGEF7B* is Expressed in Various Tissues, Predominantly in Floral Meristem and Floral Organs

**Fig. 1:**
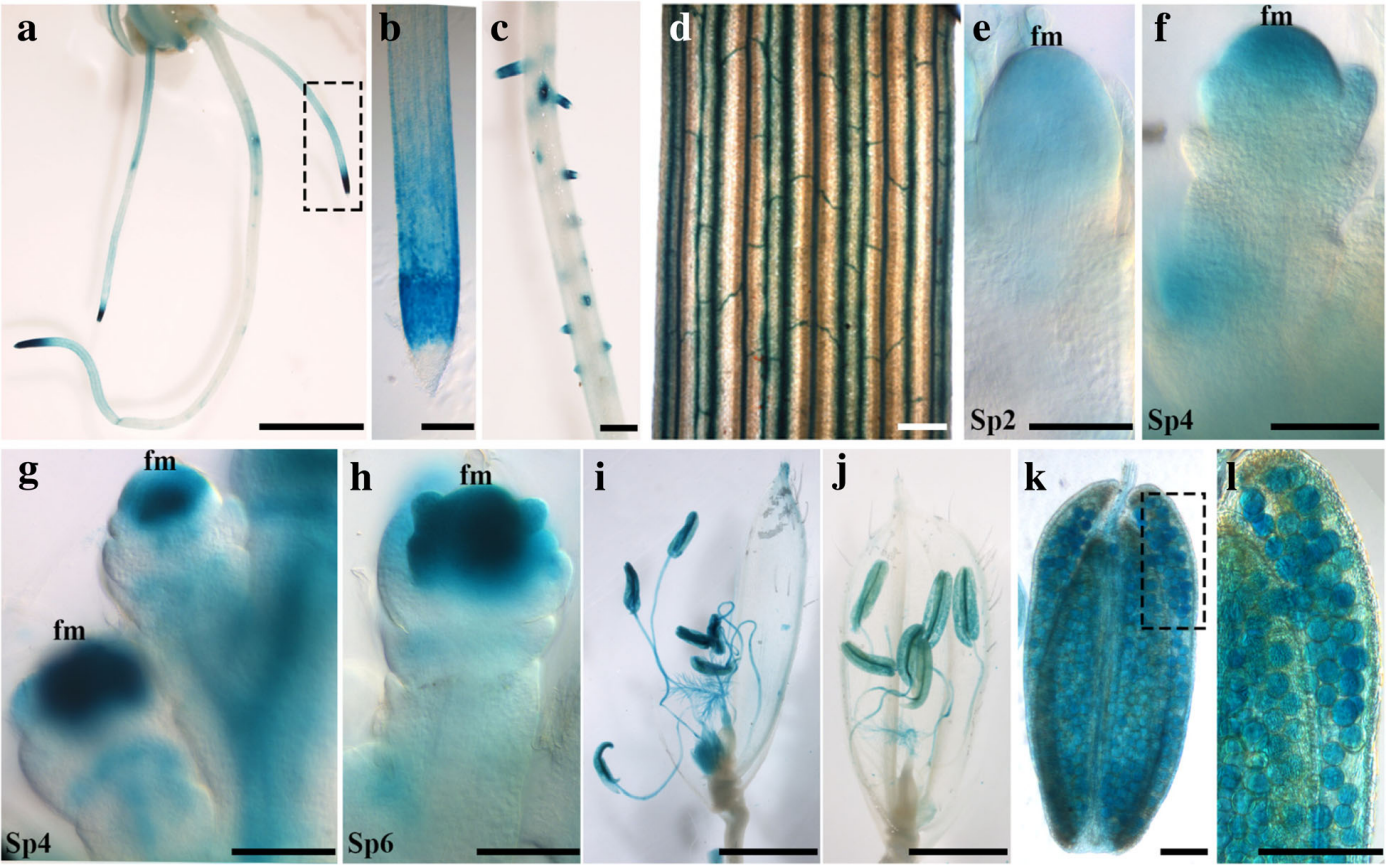
Expression profiles of *OsRopGEF7B* in rice. **a**-**d** Detection of GUS activity in 5-d-old *OsRopGEF7B*_*pro*_*:GUS* transgenic seedlings. **a** The root system. **b** Magnification of the boxed area in A. **c** Lateral root and its primordia. **d** The third leaf. **e**-**h** Analysis of GUS activity in floral meristem and floral organ primordia of different stages in *OsRopGEF7B*_*pro*_*:GUS* plants. Flower at stage Sp2 (**e**), Sp4 (**f**), later-stage Sp4 (**g**), Sp6 (**h**), according to the definition of developmental stages by Ikeda et al. (2004). (I-L) Detection of *OsRopGEF7B*_*pro*_*:GUS* expression at anthesis stages. (**i**, **j**) Whole flowers. **k** Anther. **l** Magnification of the boxed area in K. fm, floral meristem. Scale bars: (**a**) 1 cm; (**b**, **c**) 200 μm; (**d**, **e** to **h**, **k**, **l**) 100 μm; (**i**, **j**) 1 mm

To examine the expression pattern of *OsRopGEF7B*, we carried out quantitative RT-PCR (qRT-PCR) and promoter-GUS reporter gene analysis in various tissues at seedling and floral stages. qRT-PCR analysis showed that *OsRopGEF7B* was expressed in roots, stems, flag leaves, flowers, and immature seeds, specifically in flowers (Additional file 1: Figure S1). To further verify these observations, we analyzed the GUS (β-glucuronidase) activity in several *OsRopGEF7B*_*pro*_*:GUS* transgenic lines. *OsRopGEF7B* was expressed in the root of 5-day-old seedling (Fig. 1a), especially in the meristematic region of root tip (Fig. 1b). GUS activities were also detected in lateral root primordia and lateral root (Fig. 1c), and vein of the third leaf (Fig. 1d) at seedling stages. The *OsRopGEF7B* expression was also observed in the floral meristem and floral organ primordia of different stages in the transgenic plants (Fig. 1e-h), predominantly in the later-stage Sp4 (Fig. 1g) and Sp6 (Fig. 1h). When the plants reached reproductive stages, GUS activity was high in anthers, filaments and stigmas (Fig. 1i-l), most predominantly in pollen (Fig. 1l). These results are in accordance with the microarray data from the RiceXPro online databases (http://ricexpro.dna.affrc.go.jp/GGEP/graph-view.php?featurenum=3833).

### Loss-of-Function Mutation of *OsRopGEF7B* Causes Developmental Defects in Floral Organs

**Fig. 2:**
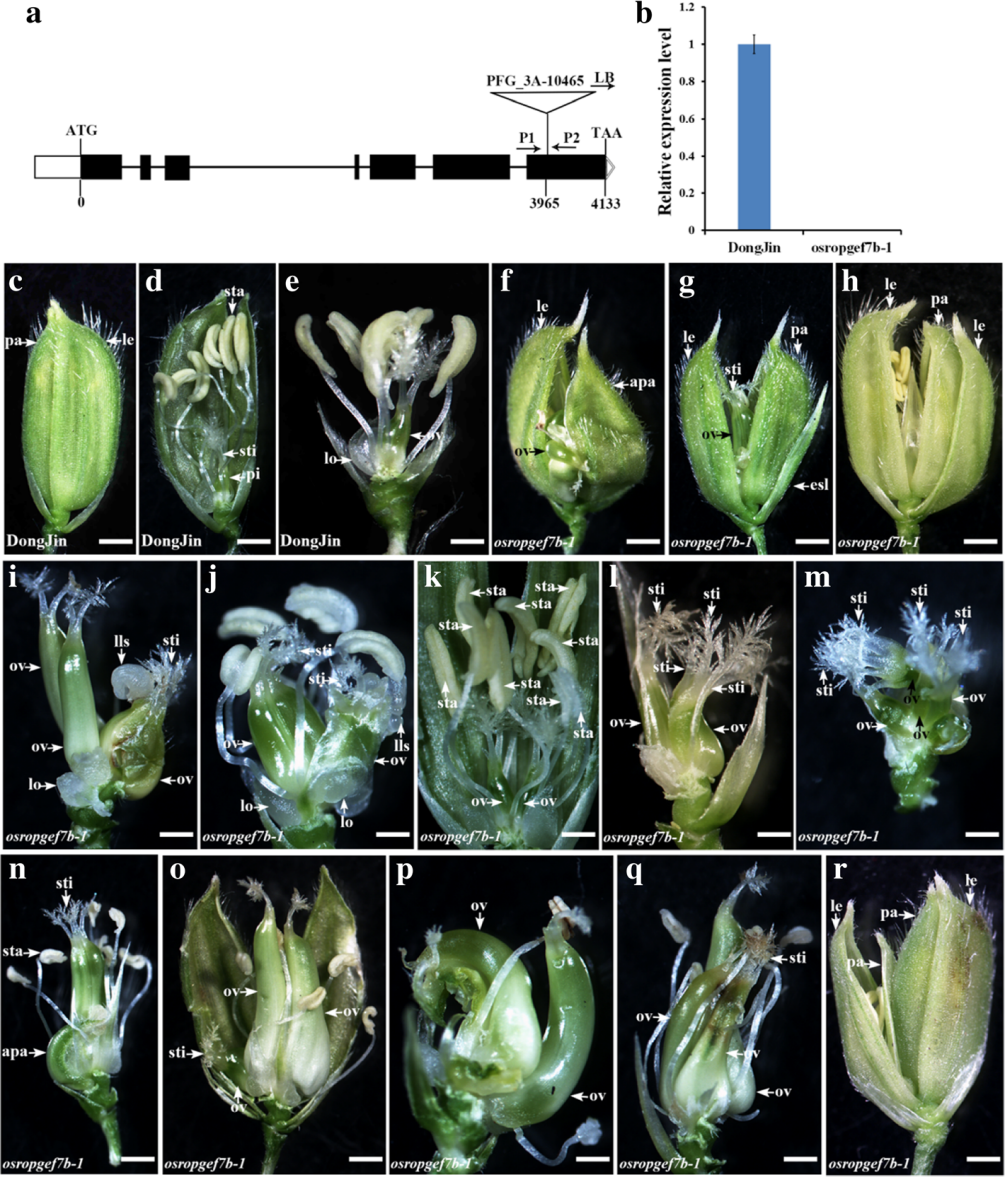
Knock-out of *OsRopGEF7B* induces severe developmental defects in floral organ. **a** Structure of *OsRopGEF7B*. Black boxes indicate exons, black thin lines between exons indicate introns, white boxes indicate UTRs. The T-DNA insertion site in the seventh exon of *osropgef7b-1*(PFG_3A-10,465) is shown and P1 and P2 indicate the sites of primers used for identifying the T-DNA insertion. **b** Transcript level of *OsRopGEF7B* in *osropgef7b-1* compared with wild type (DongJin). **c**-**r** Floral phenotypes of *osropgef7b-1* compared with wild type. Lemmas/paleae and stamens were partially or totally removed to display the inner organs. **c**-**e**) Wild type flowers. **f**-**r**) *osropgef7b-1* flowers with different phenotypes. **f** A flower with one normal lemma and one abnormal palea, and an enlarged ovary coming out from the shells (lemma/palea). **g** A flower with an elongated sterile lemma. **h** A sterile lemma transformed into a lemma structure. **i**-**j** Flowers have multiple ovaries with lodicules-like structure ectopic growth upon the top of ovary. (K) A flower with two ovaries and nine stamens. **l**-**m**) Flowers with multiple ovaries and stigmas. (**n**-**q**) Flowers with multiple fused or separated ovaries. **r** Two flowers in different sizes on a receptacle. Apa, abnormal palea; esl, elongated sterile lemma; glo, glume-like organ; le, lemma; lls, lodicules-like structure; lo, lodicules; ov, ovary; pa, palea; pi, pistil; sta, stamen; sti, stigma; Scale bars: (**c** to **r**) 1 mm

**Fig. 3:**
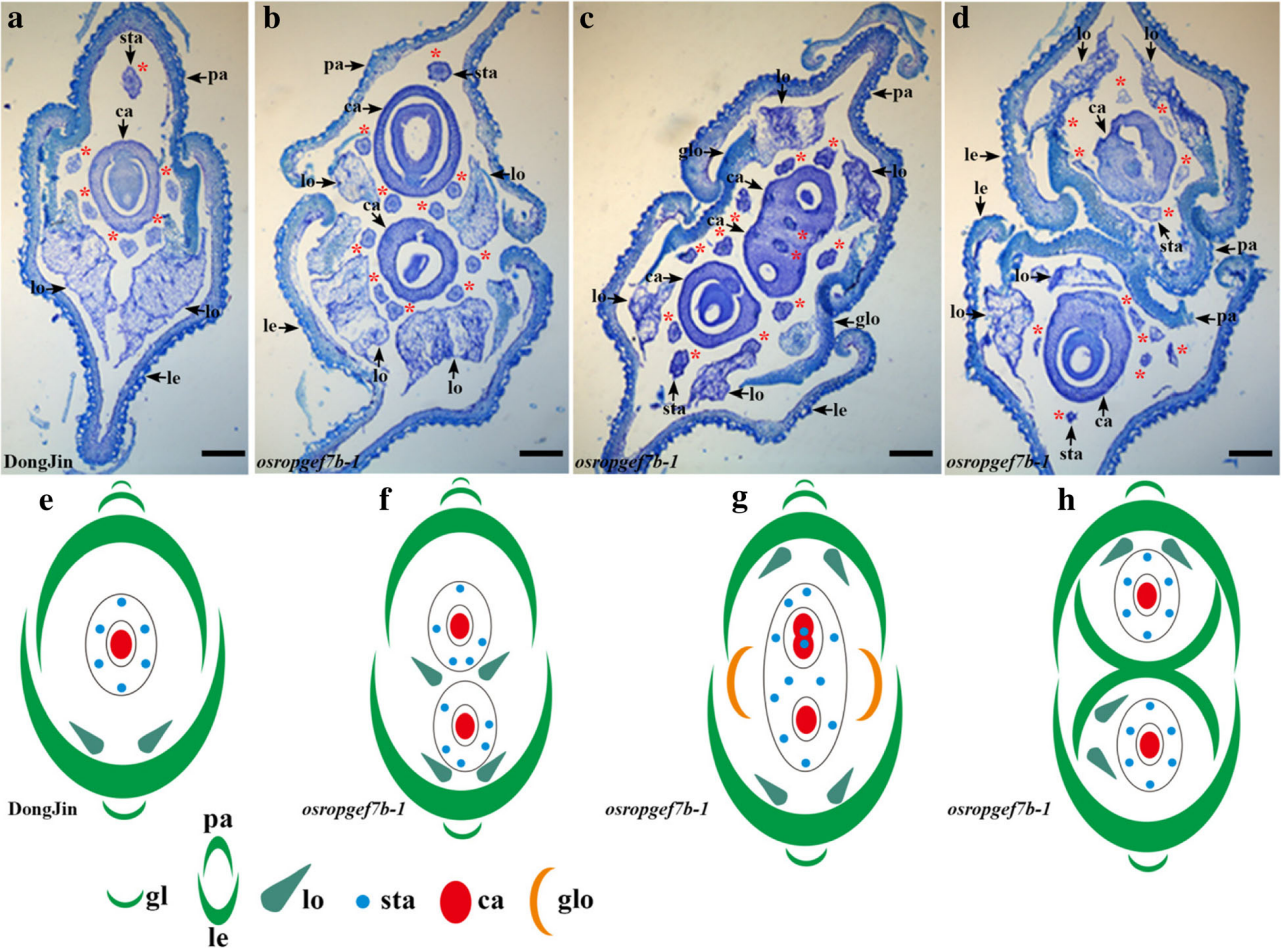
Microscopic analyses of paraffin sections of wild type and *osropgef7b-1* spikelets at basal position. **a** Transverse section of wild type. Stamens were marked with asterisks. **b**-**d** Transverse section of *osropgef7b-1*. **b** A flower with two carpels in different sizes, ten stamens and four lodicules, and non-overlapping lemma and palea. **c** A flower with three carpels, two of which were fused together, twelve stamens, two glume-like organs, four lodicules, and non-overlapping lemma and palea. **d** A flower with twelve stamens, four lodicules, and two carpels separated by two partially fused paleae. **e**-**h**) Sketches depicting the upper photos of paraffin sections, (**e**) for (**a**), (**f**) for (**b**), (**g)** for (**c**), and H for D, respectively. **e** wild type and (**f**-**h**) *osropgef7b-1* flowers. ca, carpel; gl, glume; glo, glume-like organ; le, lemma; lo, lodicules; ov, ovary; pa, palea; sta, stamen; Scale bars: (**a** to **d**) 100 μm

A T-DNA insertion line (named *osropgef7b-1*) was identified. T-DNA insertion was confirmed in the seventh exon (PFG_3A-10,465) of *OsRopGEF7B* (Fig. 2a) by PCR analysis. It was confirmed as a knock out mutant as qRT-PCR assay displayed absence of *OsRopGEF7B* transcript in this mutant (Fig. 2b). Since *OsRopGEF7B* was highly expressed in floral meristem and floral organs (Fig. 1e-l), we focused our analysis on floral development. Phenotype analysis indicated that loss of function in *OsRopGEF7B* severely affects flower organ development, 18.6% flowers (195 in 1046 flowers) showed defects (Table 1; Fig. 2f-r). In detail, the *OsRopGEF7B* mutant exhibited phenotype of a flower with an abnormal palea/lemma, an elongated sterile lemma, or homeotic transformation of a sterile lemma into a lemma structure (2.58%, *n* = 1046; Table 1; Fig. 2f-h), compared with wild type (Table 1; Fig. 2c-e). A flower has two ovaries (10.13%, *n* = 1046; Table 1; Fig. 2k, n, p) or more than two ovaries (1.34%, *n* = 1046; Table 1; Fig. 2i-j, l-m, o, q) in *osropgef7b-1* mutant, while wild type normally contains only one ovary in a flower (Fig. 2c-e). A flower with multiple ovaries always contains more than six stamens (11.7%, n = 1046; Table 1; Fig. 2k). Additionally, we observed two flowers shared with one receptacle (0.57%, *n* = 1046; Table 1; Fig. 2r). Transverse section of spikelets of *osropgef7b-1* displayed that an *osropgef7b-1* flower contains ten stamens (Fig. 3b), and the maximum could be up to 12 stamens (Fig. 3c, d), while wild type only contains six stamens (Fig. 3a). The corresponding results of transverse section of spikelets (Fig. 3a-d) were illustrated more clearly by using the diagrams (Fig. 3e-h), respectively.

**Table 1:** Numbers of floral organs in wild-type and *osropgef7b-1* plants

	Two ovaries	More than two ovaries	Multiple stigmas	Abnormal paleae/ lemmas	Two flowers	Number of stamens (*n* > 6)	Total flowers analyzed
DongJin	0	0	0	0	0	0	1151
*osropgef7b-1*	106 (10.13%)	14 (1.34%)	42 (4.02%)	27 (2.58%)	6 (0.57%)	120 (11.7%)	1046
ZH11	0	0	0	0	0	0	1420
RNAi L31	54 (3.95%)	12 (0.88%)	23 (1.68%)	11 (0.81%)	6 (0.44%)	66 (4.83%)	1366

Multiple stigmas, flower with three or more than three stigmas were counted as flower containing multiple stigmas. Flower with two or more than two ovaries always contains more than six stamens. Data were presented as mean values of three biological repeats, *n* = 1009 to 1420

### Knockdown of *OsRopGEF7B* Induces the Similar Floral Phenotypes to Those of the T-DNA Insertion Line

**Fig. 4:**
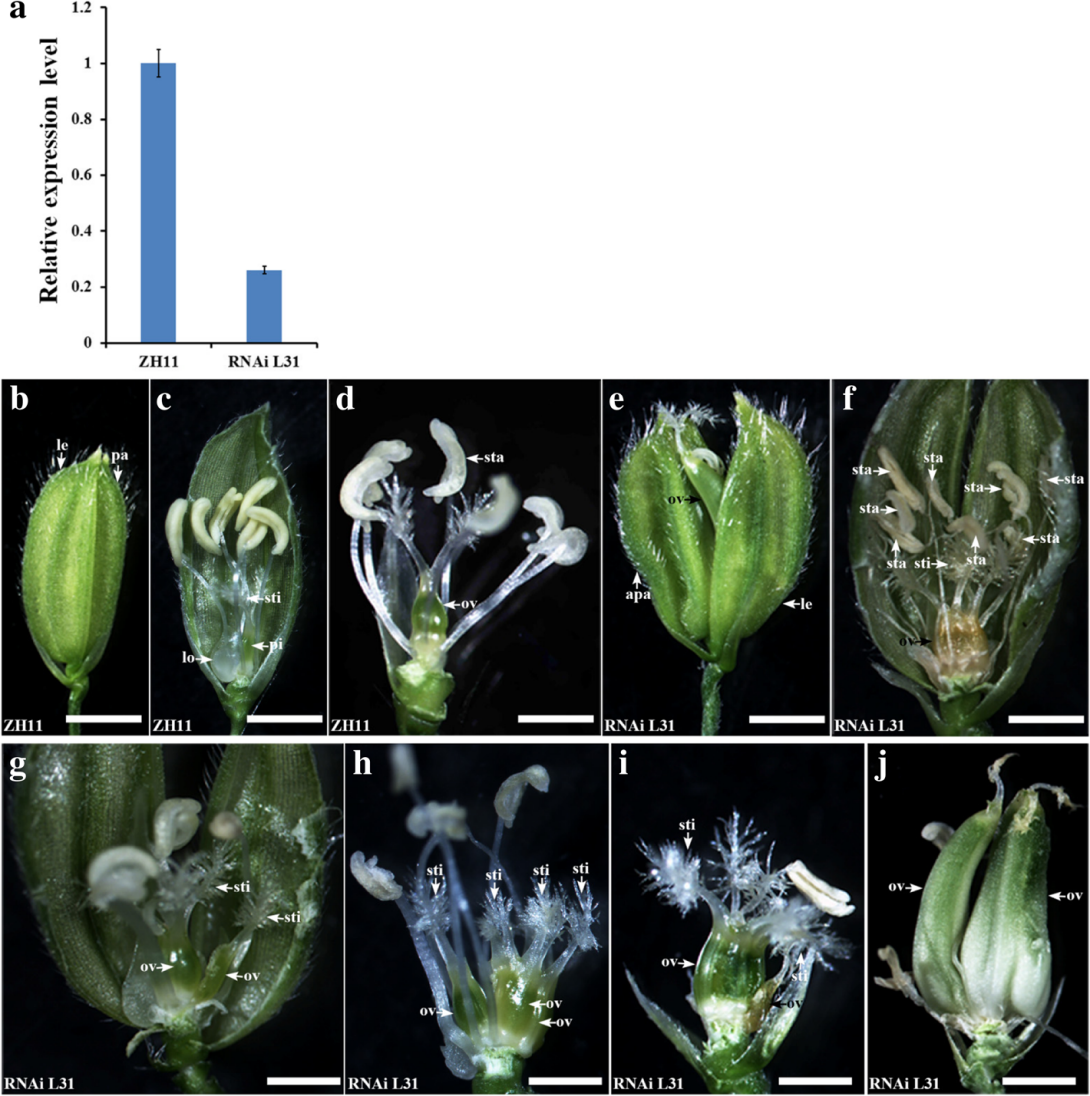
Phenotypes of flowers in RNAi L31 transgenic plants. **a** Expression levels of *OsRopGEF7B* in RNAi L31 compared with wild type (ZH11). (**b**-**j**) Floral phenotypes of RNAi L31 compared with wild type. Lemmas/paleae and stamens were partially or totally removed to show the inner organs. **b**-**d** Wild type flowers. **e**-**j** RNAi L31 flowers with various phenotypes. **e** A flower with one normal lemma and one abnormal palea, and the ovary coming out from the shells. **f** A flower with eight stamens. **g** A flower with two ovaries. **g** A flower with more than two ovaries (**i**) A flower with multiple stigmas. **j** A flower with two enlarged different sized ovaries with atrophic stigmas. Bars = 1 mm

The *osropgef7b-1* showed defects in flower development (Table 1; Fig. 2f-r). To further confirm the role of *OsRopGEF7B*, we generated the *OsRopGEF7B-RNAi* knockdown lines. In total, we obtained 40 T1 independent *OsRopGEF7B-RNAi* lines, and examined 11 lines by qRT-PCR (Additional file 1: Figure S2). The RNAi L31 with the strongest suppression in *OsRopGEF7B* mRNA levels (Fig. 4a) was chosen for subsequent analysis.

RNAi L31 showed very similar floral phenotypes as *osropgef7b-1*, in detail, nearly 8% flowers (106 in 1366 flowers) observed displayed developmental defects, including abnormal palea / lemma or elongated sterile lemma (0.81%, *n* = 1366; Table 1; Fig. 4e), more than six stamens (4.83%, *n* = 1366; Table 1; Fig. 4f), two ovaries (3.95%, *n* = 1366; Table 1; Fig. 4g, j), more than two ovaries (0.88%, n = 1366; Table 1; Fig. 4h), and multiple stigmas (1.68%, *n* = 1366; Table 1; Fig. 4i). The lower percentage of developmental defects of RNAi L31 in flower compared with *osropgef7b-1* could be explained by the fact that the transcript level of *OsRopGEF7B* in RNAi L31 still remains over 20% (Fig. 4a), but could not be detected by qRT-PCR in *osropgef7b-1* (Fig. 2b). Thus, we proposed that OsRopGEF7B plays roles in floral organ development.

### Knock-off and -down of *OsRopGEF7B* Affects Agronomic Traits in Rice

**Fig. 5:**
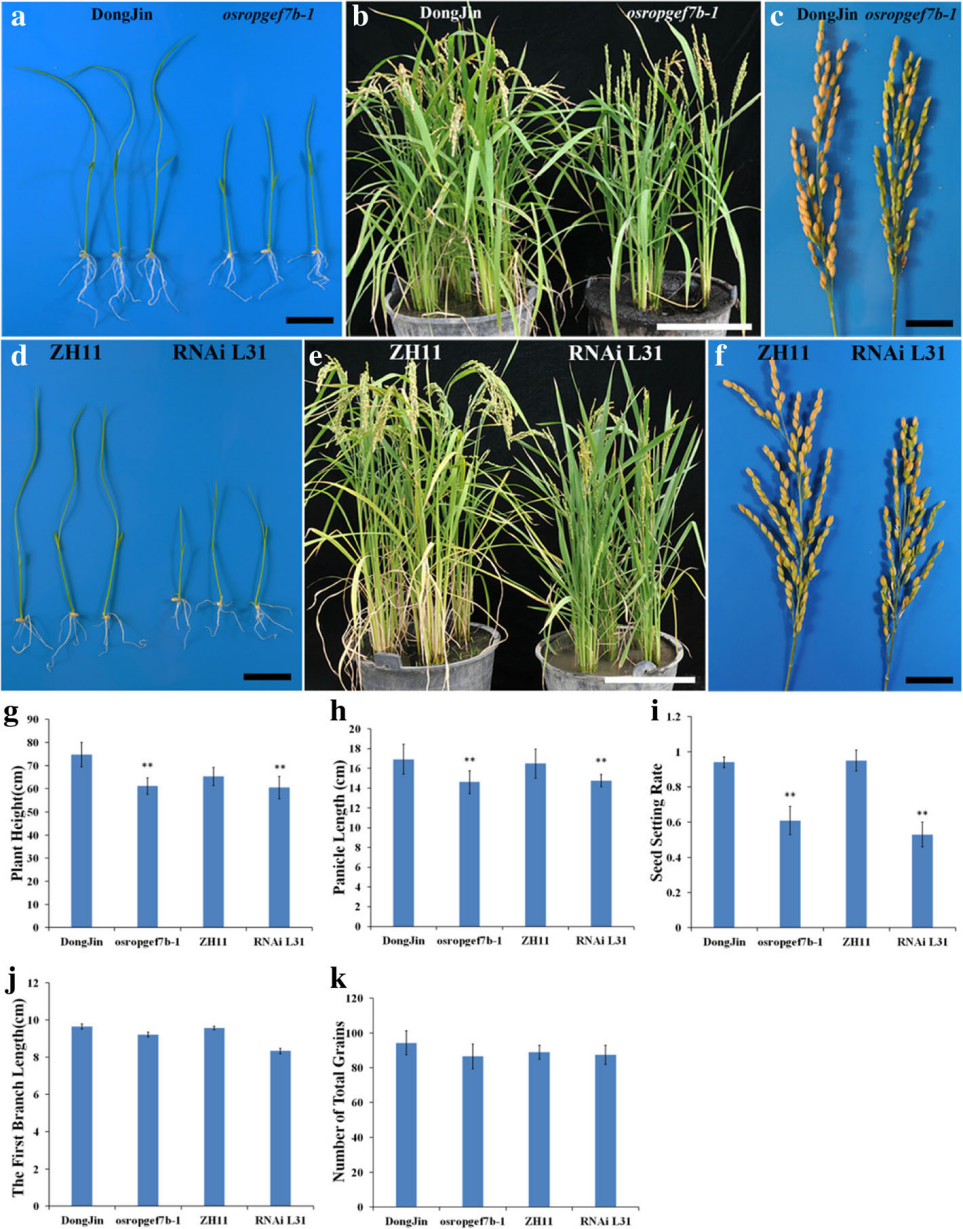
*OsRopGEF7B* affects agronomic traits in rice. (A-C) Phenotypic differences between *osropgef7b-1* and wild type. **a** 7-d-old seedling stage. **b** Mature stage. **c** Panicles. **d**-**f**) Phenotypes of RNAi L31 compared with wild type. **d** 7-d-old seedling stage. **e** Mature stage. **f** Panicles. **g**-**k** Main agronomic traits were influenced by the knock-out or knock-down of *OsRopGEF7B* in *osropgef7b-1* and RNAi L31 compared with wild type. **g** Plant height. **h** Panicle length. **i** Seed setting rate. **j** Length of first branch. **k** Number of total grains per panicle. Data were presented as mean values of three biological replicates with SD, *n* = 60 to 80. Scale bars: (**a**, **c**, **d**, **f**) 5 cm; (**b**, **e**) 30 cm

Since the *osropgef7b-1* mutant and RNAi L31 transgenic plant displayed similar floral phenotypes (Table 1; Figs. 2f-r, 4e-j), we further analyzed their agronomic traits. Plant height of both, *osropgef7b-1* and RNAi L31, was markedly reduced in 7-day-old seedlings (Fig. 5a, d) and even in mature stage (Fig. 5b, e, g), compared with wild type. Panicles length and seed setting rate were notably reduced in both *osropgef7b-1* and RNAi L31 relative to the control (Fig. 5c, f, h, i). In addition, both of the first branch length (Fig. 5j) and the numbers of total grain per panicle (Fig. 5k) slightly decreased, compared with their controls.

Because *OsRopGEF7B* was highly expressed in pollen (Fig. 1k, l), it likely plays roles in pollen development similar to the homolog in Arabidopsis, *AtRopGEF12*. Overexpression of a C-terminally truncated AtRopGRF12 interrupted pollen tube growth (Zhang and McCormick 2007). Thereofore, we analyzed the pollen development of *osropgef7b-1* and RNAi L31, and our observations indicated that the percentage of pollen fertility from *osropgef7b-1* mutant and RNAi L31 plant (Additional file 1: Figure S3B, D, I) are same to those of the wild type controls (Additional file 1: Figure S3A, C, I). Results of DAPI staining showed that all the fertile pollen grains from *osropgef7b-1*, RNAi L31 and the wild type contained three nuclei: two bright, intensely stained sperm nuclei and one diffused, weakly stained vegetative nucleus (Additional file 1: Figure S3E-H). The DAPI analysis indicated that the knock-out or -down of *OsRopGEF7B* did not affect pollen development. We also found that pollen tube elongation and pollen germination in vivo of both *osropgef7b-1* mutant (Additional file 1: Figure S4D-F, M) and RNAi L31 plant (Additional file 1: Figure S4J-M) were not different from those of the wild type (Additional file 1: Figure S4A-C, G-I, M), indicating that the knock-out or -down of *OsRopGEF7B* did not affect pollen tube elongation. Taken together, our data suggest that the reduced seed setting of *osropgef7b-1* mutant and RNAi plants might be partly attributable to the defects in female organ development, as pollen development and pollen tube growth in *osropgef7b-1* and RNAi plants are quite normal.

### *OsRopGEF7B* Does Not Affect the Expression of a Set of Transcription Factors Which are Associated with Floral Development in Rice

To further investigate how *OsRopGEF7B* works in regulating flower development, we examined several genes which encode transcription factors associated with floral development. Genetic studies showed that mutation in *YABBYs*, *ETTIN*, *OsMADS1*, *OsMADS6* and *OsMADS55* affect floral organ identification (Sessions et al. 1997; Nemhauser et al. 2000; Prasad et al. 2005; Yadav et al. 2011; Teo et al. 2014). Therefore, we carried out qRT-PCR to analyze the transcripts of these genes in both *osropgef7b-1* and RNAi L31 at both seedling and floral stages. The results indicated that the genes analyzed were not significantly affected by *OsRopGEF7B* mutation (Additional file 1: Figure S5A, B).

We previously reported that knock-down of RopGEF7 affects PIN1 accumulation and polarization to impact polar auxin transport and thereby influences embryo and root development in *Arabidopsis* (Chen et al. 2011). PIN1 is also required for floral development (Yamaguchi et al. 2013; Holt et al. 2014). Thus we performed the qRT-PCR analysis of four *OsPINs*, and the data showed no changes in neither of them compared with wild type at both seedling and floral stages (Additional file 1: Figure S5C, D). Our data suggest that *OsRopGEF7B* might do not influence the expression of *YABBYs*, *ETTIN*, *OsMADS1*, *OsMADS6*, *OsMADS55* and *PIN1* in rice.

### OsRopGEF7B interacts with OsRACs in Rice Protoplasts and Yeast Cells

**Fig. 6:**
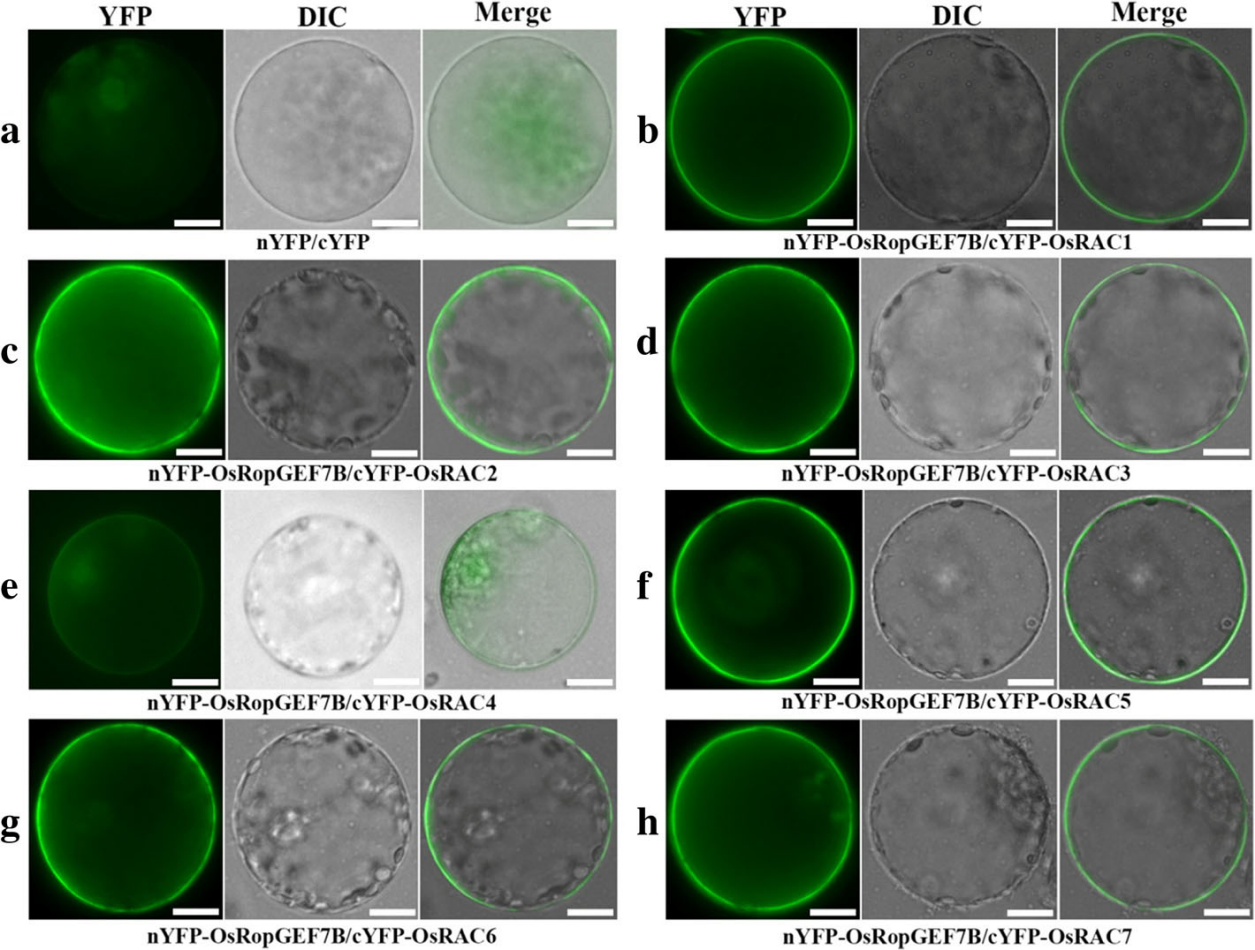
OsRopGEF7B interacts with OsRACs in rice protoplasts. Rice protoplasts were co-transfected with (**a**) N-terminal *YFP* and C-terminal *YFP*, (**b**) *nYFP-OsRopGEF7B* and *cYFP-OsRAC1*, (**c**) *nYFP-OsRopGEF7B* and *cYFP-OsRAC2*, (**d**) *nYFP-OsRopGEF7B* and *cYFP-OsRAC3*, (**e**) *nYFP-OsRopGEF7B* and *cYFP-OsRAC4*, (**f**) *nYFP-OsRopGEF7B* and *cYFP-OsRAC5*, (**g**) *nYFP-OsRopGEF7B* and *cYFP-OsRAC6*, (**h**) *nYFP-OsRopGEF7B* and *cYFP-OsRAC7*. Images were acquired under the YFP and differential interference contrast (DIC) channel, respectively, and then merged. Bars = 10 μm

**Fig. 7:**
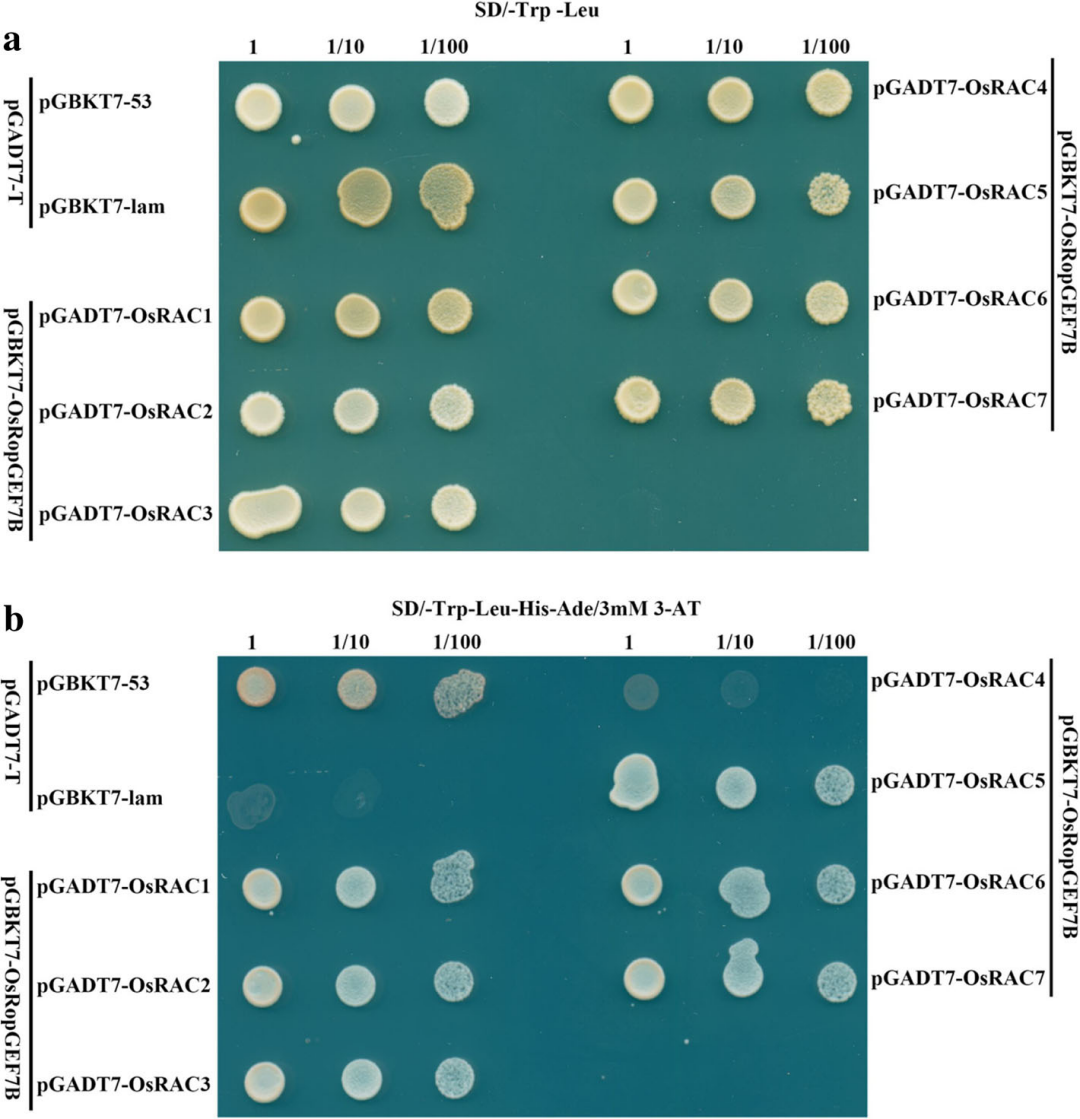
Interactions between OsRopGEF7B and OsRACs in the Y2H assay. The transformants were plated onto (**a**) double dropout medium (SD/−Trp/−Leu) and onto (**b**) quadruple dropout medium (SD/−Trp/−Leu/-His/−Ade) supplemented with 3-AT. The pairs of pGBKT7–53 / pGADT7-T and pGBKT7-Lam / pGADT7-T were used as positive and negative controls, respectively. The growth of co-transformed yeast cells in the quadruple dropout medium supplemented with 3-AT is indicative of protein–protein interaction in yeast

Biochemical, structural and functional studies suggest that *Arabidopsis* RopGEFs act as activators of ROP/RACs (Berken et al. 2005; Thomas et al. 2007; Craddock et al. 2012; Bloch and Yalovsky 2013; Chang et al. 2013; Yalovsky 2015). To identify the relationship between *OsRopGEF7B* and *RAC/ROPs*, we carried out the bimolecular fluorescence complement (BiFC) analysis in the plant cells. When BiFC constructs of *OsRopGEF7B* in combination with seven individual *OsRACs* were cotransformed into rice protoplasts, BiFC-generated apparent yellow fluorescent protein (YFP) signal in the plasma membrane was observed (Fig. 6b-d, f-h) except for OsRAC4 which only shows very weak YFP signal (Fig. 6e). The controls displayed only background signal (Fig. 6a), indicating that OsRopGEF7B interacted with all of seven OsRACs in plasma membrane in rice protoplasts. To further identify the interactions between OsRopGEF7B and OsRACs, we performed the yeast two-hybrid (Y2H) assay. The results suggested that OsRopGEF7B strongly interacts with six OsRACs but only weakly with OsRAC4 (yeast growth seen only after seven days, Additional file 1: Figure S6B) on quadruple dropout medium supplemented with 3 mM 3-AT, compared to both positive and negative controls (Fig. 7; Additional file 1: Figure S6B). The BiFC and Y2H analyses suggested that OsRopGEF7B strongly interacts with six OsRACs but only very weakly with OsRAC4. Taken together, our data suggest that OsRopGEF7B may activate OsRACs through direct interaction in the signaling pathway, to regulate floral organs development in rice.

## Discussion

The roles of RopGEFs in regulating plant growth and development in rice were rarely reported. Here, we provided experimental evidence that *OsRopGEF7B* has functions during the vegetative growth and reproductive development in rice, especially in the processes of floral organ development.

Our data revealed that *OsRopGEF7B* is predominantly expressed in floral meristem, floral organ primordia, anther, filament and stigma (Fig. 1e-l). The knock-out mutant and knock-down transgenic plants, *osropgef7b-1* and RNAi L31, respectively, showed increases in the numbers of the inner floral organs (ovary and stamen) (Table 1; Figs. 2i-q, 4f-j). Specifically, some of the *osropgef7b-1*(10.13%) and RNAi L31 (3.95%) plants have more than one ovary (Table 1; Figs. 2i-q, 4f-j). In the wild type flower, carpel is composed of ovary, style and stigma; the carpel primordium is developed from the floral meristem (Chu et al. 2006). Initiation of the floral meristems is the start of flower development, followed by floral meristem identity specification and maintenance, floral organ primordia initiation, floral organ identity specification, floral stem cell termination, and finally floral organ maturation (Guo et al. 2015). In this multistep process, each of numerous genes is expressed in a spatiotemporally regulated manner. Phenotypes of mutants give clues which role the corresponding gene plays in flower development. The *floral organ number1* (*fon1*) mutant of *Arabidopsis* shows abnormal flowers from stage 6, after the three-whorl stamen primordia have initiated. The prolonged floral meristem activity continues to produce extra stamen and carpel primordia, resulting in generating additional stamens and carpels (Huang and Ma, 1997). The *fon1* mutant of rice also exhibits an enlarged floral meristem and subsequently increased the number of all floral organs (Nagasawa et al., 1996; Suzaki et al., 2004). *FON4* of rice plays a more important role on the carpel development than on the outer whorls; the *fon4–1* displayed an increase in the number of carpel that results from the extra carpel primordia in the enlarged floral meristem; almost all *fon4–1* and *fon4–2* flowers and most of *fon4–3* flowers contain more than one carpel (Chu et. 2006). Thus, phenotypic similarities between *osropgef7b-1* (and RNAi L31) and *fon* mutants suggest that mutation in *OsRopGEF7B* might strengthen the activity of flower meristem subsequently producing extra carpel primordia resulting in the generation of additional carpels (as well as additional ovaries). Thus, normal expression of *OsRopGEF7B* seems to be required for restricting the numbers of inner floral organs. To understand the mechanism underlying this phenotype, the detailed morphological analysis of the floral meristem in *osropgef7b-1* and RNAi L31 plants compared to wild type is needed in the future study.

Previously, our group reported that *Arabidopsis* RopGEF7, grouped into the same clade with OsRopGEF7B (Additional file 1: Figure S7), maintains the stem cell niche by auxin-dependent PLT pathway. Knock-down of *AtRopGEF7* by RNA interference technique indicated that AtRopGEF7 can regulate the auxin efflux transporter PIN1, PIN3 and PIN7 accumulation and thereby affects polar auxin transport that affects root development (Chen et al. 2011; Huang et al. 2014). Additionally, auxin plays vital roles in determining floral organ development. Plants harboring mutations in *PIN1* often show strong defects in inflorescence with naked stems, often produce only a few abnormal flowers. *pin1* mutant flowers exhibit variable petals number, fused floral organs, and expansion of the stylar and stigmatic regions of the gynoecium (Okadala et al. 1991; Cheng and Zhao 2007; Yamaguchi et al. 2013; Holt et al. 2014). Similarly, *osropgef7b-1* and RNAi L31 exhibited fused ovaries (Table 1; Figs. 2i-q, 4f-j), and *osropgef7b-1* showed three glumes (Fig. 2h). Though our results indicated that the transcripts of all four *OsPIN1* members are maintained at the same levels in *osropgef7b-1* and RNAi L31 as those in the wild type (Additional file 1: Figure S5C, D), we could not exclude the possibility that OsRopGEF7B might regulate PIN1 at the posttranscriptional level.

In *Arabidopsis*, overexpression of RopGEF1 and RopGRF12 affects pollen tube growth (Gu et al. 2006; Zhang and McCormick 2007). Meanwhile, the microarray data from the RiceXPro online database (http://ricexpro.dna.affrc.go.jp/category-select.php) showed that nine out of eleven rice *RopGEFs* were highly expressed in anther. The *OsRopGEF7B*_*pro*_*:GUS* analysis results indicate that *OsRopGEF7B* is strongly expressed in anther (Fig. 1i-l). However, our data indicated that knock-out or -down of *OsRopGEF7B* does not affect pollen development (Additional file 1: Figure S3) and pollen tube elongation (Additional file 1: Figure S4), which might be explained by the functional redundancy of *OsRopGEF* members. Interestingly, we also found that knock-out or -down of *OsRopGEF7B* markedly influenced plant height, panicle length, and seed setting rates in *osropgef7b-1* and RNAi L31 in comparison with wild type (Fig. 5a-i). Taken together, these results suggest that *OsRopGEF7B* influences rice seed setting partly through mediating floral organ development.

The X-ray structure analysis provided that the catalytic PRONE domain of AtRopGEF8 is found in a ternary complex with Rop4 and GDP (Thomas et al., 2007) and immunity studies have provided evidence that PRONE-type OsRacGEFs may be involved in disease responses through activation of OsRAC1 in rice (Kawasaki et al. 2009; Kawano et al., 2010), and OsRopGEF10 regulates small papillae development through activating OsRAC1 (Yoo et al. 2011). Additionally, yeast two-hybrid assays displayed that OsRAC1 interacts with OsRacGEF1 and OsRacGEF2 via their PRONE domain (Akamatsu et al. 2013, 2015), and the CEBiP/CERK1-OsRacGEF-OsRac1 module plays a major role for early signaling in rice chitin-triggered immunity (Akamatsu et al. 2013). The above-mentioned evidence suggested that OsRopGEFs act at the upstream of OsRACs. Given the interactions between RopGEFs and RACs during various biological processes in the plant cells, we further investigated the relationship between RopGEFs and RACs in rice protoplasts and yeast cells. Expectedly, the BiFC analysis indicated that OsRopGEF7B interacts with all OsRACs at plasma membrane but OsRAC4 which only shows very weak interaction (Figure 6), and the Y2H assay confirmed the results of the BiFC analysis (Figure 7; Additional file 1: Figure S6B). Therefore, it is speculated that *RopGEF7B* might interact with OsRACs in plant cells. Identifying the exact cooperative roles of OsRopGEF7B and OsRACs will help to further elucidate how *RopGEF7B* regulates rice floral development.

## Conclusions

*OsRopGEF7B* is predominantly expressed in floral organs, especially in meristem, floral organ primordia, anther, filament and stigma. *osropgef7b-1* mutant and RNAi L31 transgenic plants, exhibited increase in the numbers of the inner floral organs (ovary and stamen), generating decrement in seed setting. Meanwhile, OsRopGEF7B strongly interacted with OsRACs except the OsRAC4 which only shows very weak interaction. It suggested that OsRopGEF7B mediates the development of floral organs via activating OsRACs in rice. Understanding the function of *OsRopGEF7B* gives new clues to further elucidate the development of rice floral organs and has possible application in genetically modified crops.

### Methods

#### Plant Materials and Growth Conditions

Rice plants (*Oryza sativa* cv. Zhonghua11 (ZH11), DongJin, RNAi lines, and *osropgef7b-1*) were grown under normal field conditions in the rice growing season at the South China Agricultural University, Guangzhou, or grown in a growth chamber under 14-h-light long-day conditions at 28 °C day/night cycles.

#### Chimeric Gene Construction and Plant Transformation

The *OsRopGEF7B*_*pro*_*:GUS* construct was generated by inserting a 3024 bp *OsRopGEF7B* promoter sequence into pCAMBIA 1305.1 at *Pst*I and *Nco*I sites. To construct the RNAi vector, a 327 bp fragment of *OsRopGEF7B* was amplified from the cDNA pool using the primer set *OsRopGEF7B-RNAi*-F and *OsRopGEF7B-RNAi*-R, then inserted into the *Bam*HI and *Hin*dIII sites (for forward insertion) and the *Mlu*I and *Pst*I sites (for reverse insertion) of the pYLRNAi.5 vector with an Ubiquitin promoter provided by Dr. Yao-Guang Liu (Luo et al. 2013). These binary constructs were introduced into *Agrobacterium tumefaciens* strain EHA105 and were used for rice transformation following a previously described protocol (Li et al. 2011; Liu et al. 2014). Primers for generating these constructs were listed in Additional file 2: Table S1.

#### RNA Isolation and qRT-PCR Analyses

For analyzing gene expression, total RNA was extracted using the RNeasy plant mini kit (Qiagen). Genomic DNA contamination was removed by RNase-free DNase I treatment. First-strand cDNA was synthesized with Primescript cDNA synthesis kit (TaKaRa) and oligo (dT) primers. qRT-PCR analysis was performed using the synthesized cDNAs and the primers listed in Additional file 2: Table S1. Constitutively expressed *OsActin1* (accession No. LOC_Os03g50885) was used as an internal control to which the level of *OsRopGEF7B* in different tissues was normalized. Data are presented as averages with SD from at least three biological replicates.

#### Paraffin Section and Microscopy

Rice spikelets were fixed in a fixative solution (50% ethanol, 10% glacial acetic acid and 3.7% formaldehyde) overnight at 4 °C. After dehydration with ethanol in a series of concentrations and infiltration with xylene, the materials were embedded in paraffin (Sigma-Aldrich) as described before (Zhang et al. 2010). Sections of 8 μm in thickness were cut with a microtome and stained with 0.05% toluidine blue. Samples were observed using Nomarski optics on an Olympus BX51 microscope connected to a Ritiga 2000R digital camera.

#### Pollen in Vivo Germination and Elongation Analyses

To examine the pollen grains, rice mature flowers 1 day prior to anthesis were collected and fixed in 70% (*v*/v) ethanol and then the pollen grains were stained with I_2_-KI staining as described by Huang et al. (2014). The pollen grains were counted under a bright field microscope (Olympus BX51) and densely stained pollen grains were counted as fertile pollen grains. For 4′,6-diamidino-2-phenylindole (DAPI) staining, pollen grains from the dehisced anthers were fixed in 3:1 ethanol:acetic acid (EAA) solution for 1 h, then dehydrated and stained with DAPI. The stained pollen grains were observed using a microscope under UV light (Leica DM2500).

To analyze the pollen in vivo germination, rice flowers were emasculated and artificially pollinated. After 30 min, the pistils were collected and directly stained with aniline blue on a glass slide for 2–3 min before observation by fluorescence microscopy (Olympus BX51) as described by Chhun et al. (2007). The pollen tube growth of germinated pollen attached to stigmas was visualized by fluorescence microscopy with CFP channel.

To assay pollen in vivo elongation, at 30 min, 1 h, and 2 h time points following artificial pollination, rice pistils were collected and fixed in EAA solution for 30 min and then softened in 1 N KOH for 30 min at 55 °C. Next, the pistils were washed with PBS buffer for 2 h at room temperature. The samples were observed by fluorescence microscopy (Olympus BX51) with CFP channel. Pollen elongation can be seen in a time-dependent manner (Chhun et al. 2007).

#### Bimolecular Fluorescence Complement (BiFC) Assay in Rice Protoplasts

To generate the fusion proteins of nYFP-OsRopGEF7B and cYFP-OsRACs, the coding sequences of *OsRopGEF7B* and *OsRACs* were inserted into the *EcoR*I and *Sal*I sites of pSAT6-nEYFP-C1 and pSAT6-cEYFP-C1 vectors (Citovsky et al. 2006), respectively. Both plasmids of *35S*_*pro*_*:nYFP:RopGEF7B*, and *35S*_*pro*_*:cYFP:OsRACs* were cotransformed into rice protoplasts for BiFC assays. Protoplast isolation and transfection were performed as described by Tao et al. (2005). YFP was visualized by an Olympus BX51 microscope using YFP filter sets: the excitation and emission filters Ex490 to 510 nm/DM515 nm/BA520 to 550 nm (Tao et al. 2005). Primers used for constructing the above-mentioned plasmids were listed in Additional file 2: Table S1 online.

#### Yeast Two-Hybrid Assay

The full-length coding sequences of *OsRopGEF7B* and *OsRACs* were amplified and cloned into pGBKT7 (BD, Clontech) and pGADT7 (AD, Clontech) vectors, respectively. The primers used to generate the Y2H constructs were listed in Additional file 1: Table S1. The constructs were co-transformed by pairs into the yeast stain AH109, and the transformants were selected on synthetic dextrose (SD) medium lacking tryptophan and leucine (SD/−Trp/−Leu) following incubation at 28 °C for 3~ 4 days according to the Yeast Protocol Handbook (Cat. No. 630412, Clontech, Japan). Single co-transformed yeast clones of SD/−Trp/−Leu plates were transferred to SD medium lacking tryptophan, leucine, histidine and adenine (SD/−Trp/−Leu/-His/−Ade) in 10-fold serial dilutions to identify the protein-protein interactions. The positive (pGBKT7–53 / pGADT7-T) and negative (pGBKT7-Lam / pGADT7-T) controls were used. After confirmation that neither autoactivation nor toxicity exists in those yeast cells co-transformed with pGBKT7-OsRopGEF7B and the null pGADT7 vector as well as pGADT7-OsRACs and the null pGBKT7 vector, respectively, the Y2H screening was performed according the procedure in the Yeast Protocol Handbook. The potential protein-protein interactions were confirmed by growing the yeast cells in SD/−Trp/−Leu/-His/−Ade/3-AT plates supplemented with 3 mM 3-AT (3-amino-1,2,4-triazole, Sigma-Aldrich).

#### Statistics

All the data represented the average with the standard deviation of the average (SD) from three biological experiments. Significant difference was determined by paired two-tailed Student’s *t*-tests. *P* < 0.05 was considered significant.

## Additional files

Additional file 1: **Figure S1.** Relative expression levels of *OsRopGEF7B* in various tissues of rice at vegetative and reproductive stages. **Figure S2.** Relative expression levels of *OsRopGEF7B* in *OsRopGEF7B-RNAi* lines. **Figure S3.** *OsRopGEF7B* does not affect pollen development. **Figure S4.** In vivo pollen germination and PT elongation. **Figure S5.** Relative expression levels of a subset of genes associated with floral development in *osropgef7b-1* mutant and RNAi L31 line at both seedling and floral stages. **Figure S6.** Interactions between OsRopGEF7B and OsRACs in the Y2H assay after seven days of growth. **Figure S7.** Phylogenetic relationships between OsRopGEFs and AtRopGEFs. (PPTX 4833 kb)

Additional file 2: **Table S1.** List of primers used in this study. (DOCX 20 kb)
