# Supplementary figures and images for: Guanine Nucleotide Exchange Factor 7B (RopGEF7B) is involved in floral organ development in Oryza sativa
Source: Rice (N Y). 2018 Jul 30;11:42. doi: 10.1186/s12284-018-0235-0 (PMC6066601; doi:10.1186/s12284-018-0235-0)

## Slide 1
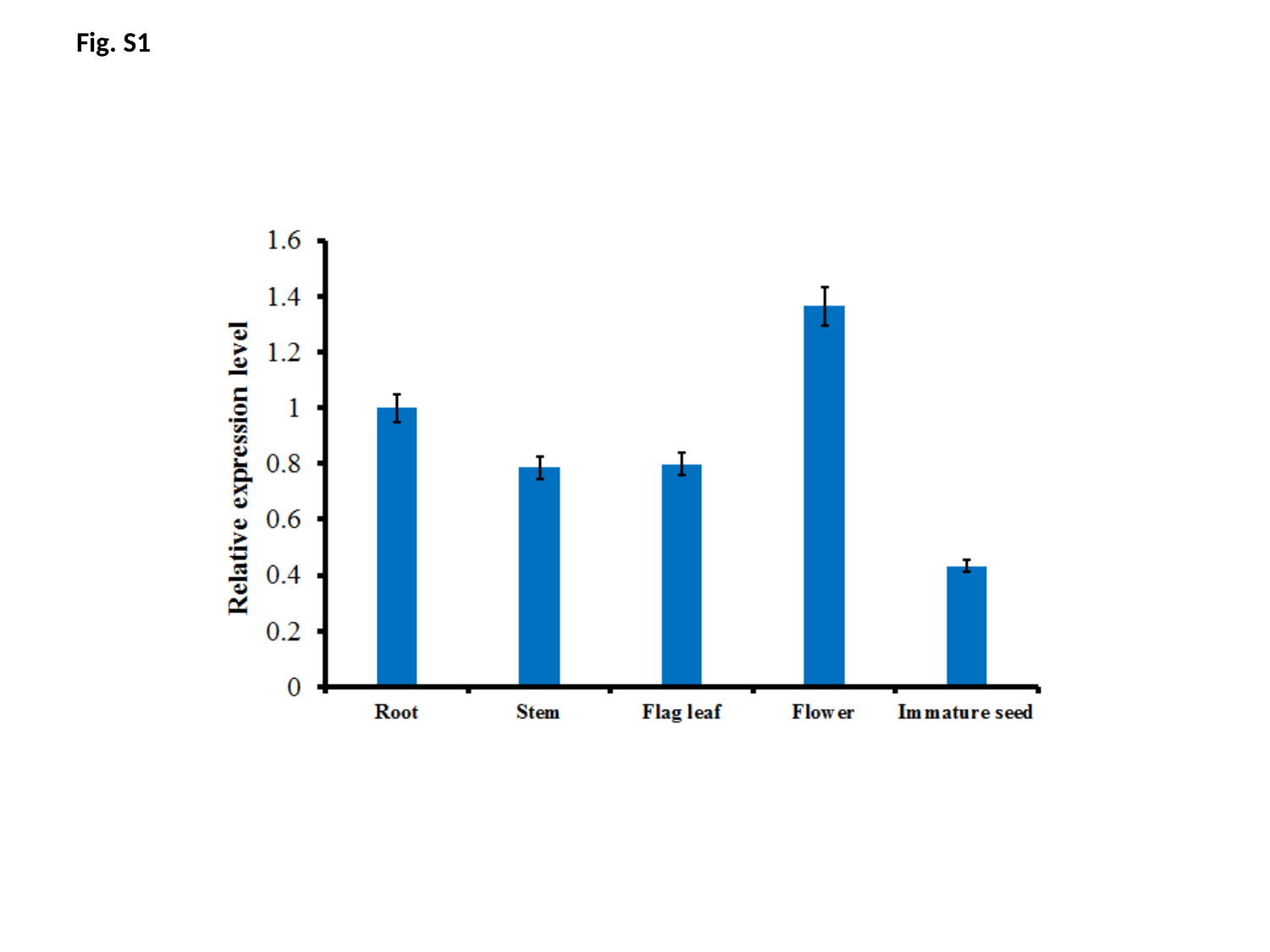

Fig. S1

## Slide 2
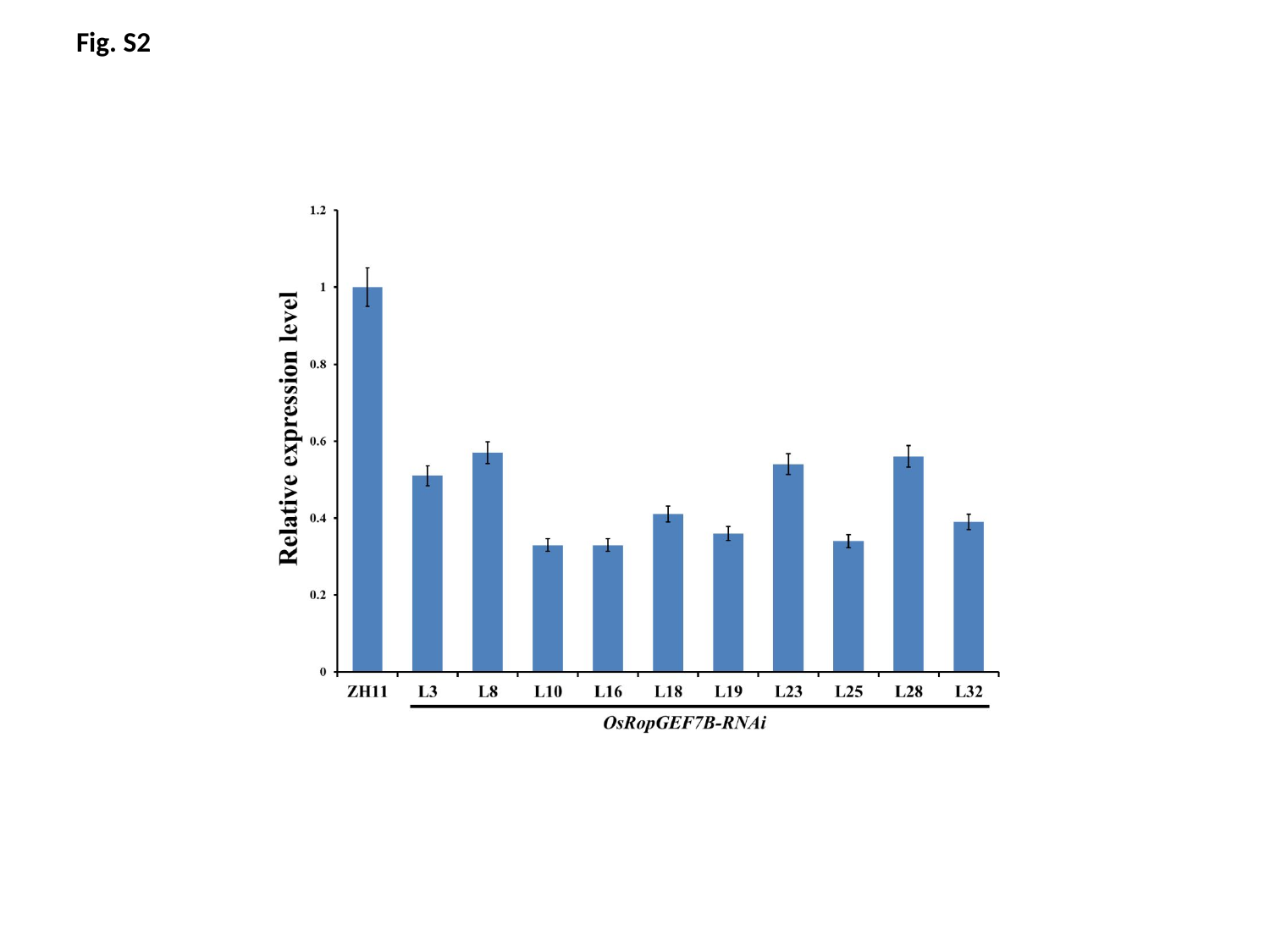

Fig. S2

## Slide 3
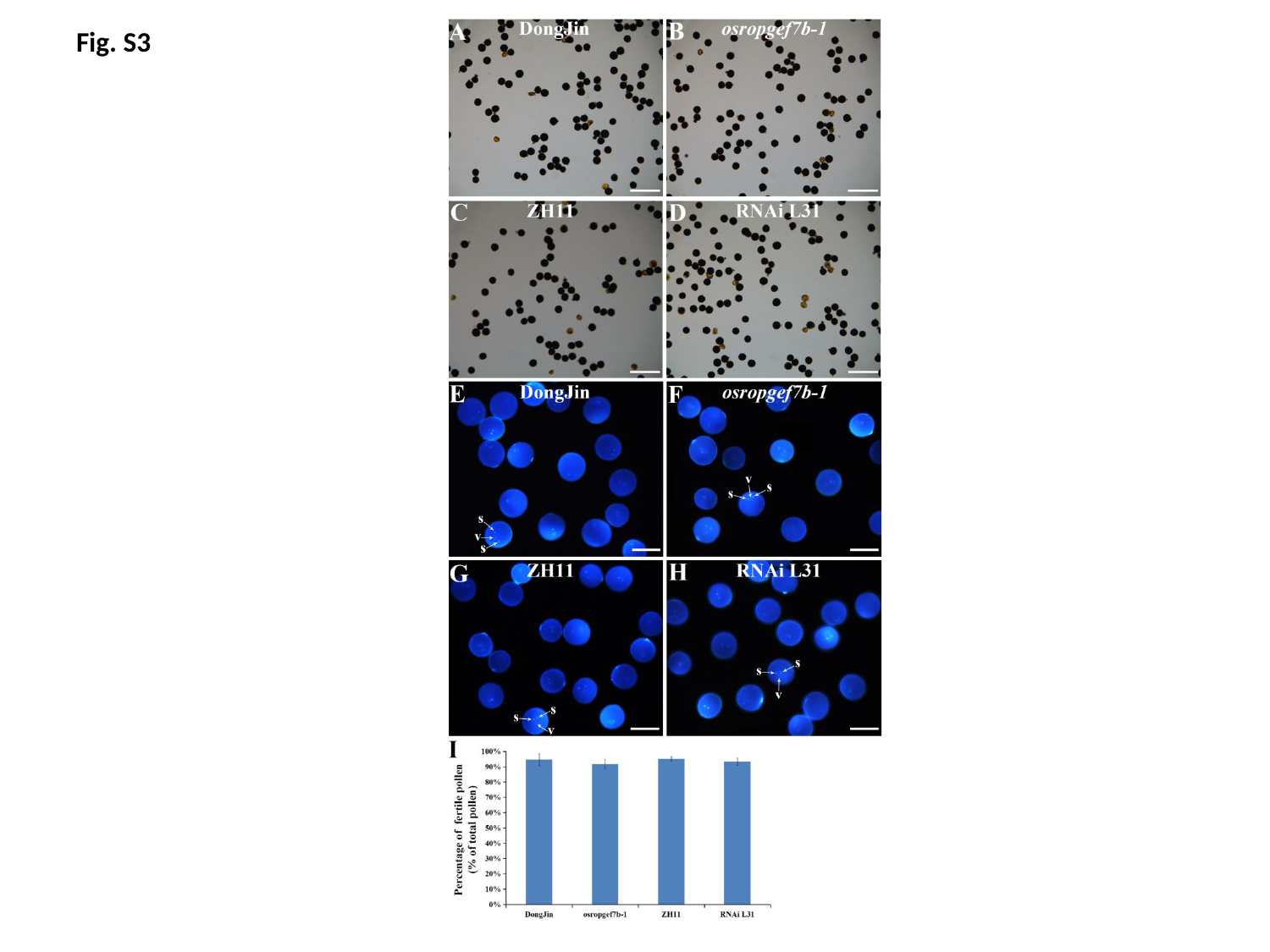

Fig. S3

## Slide 4
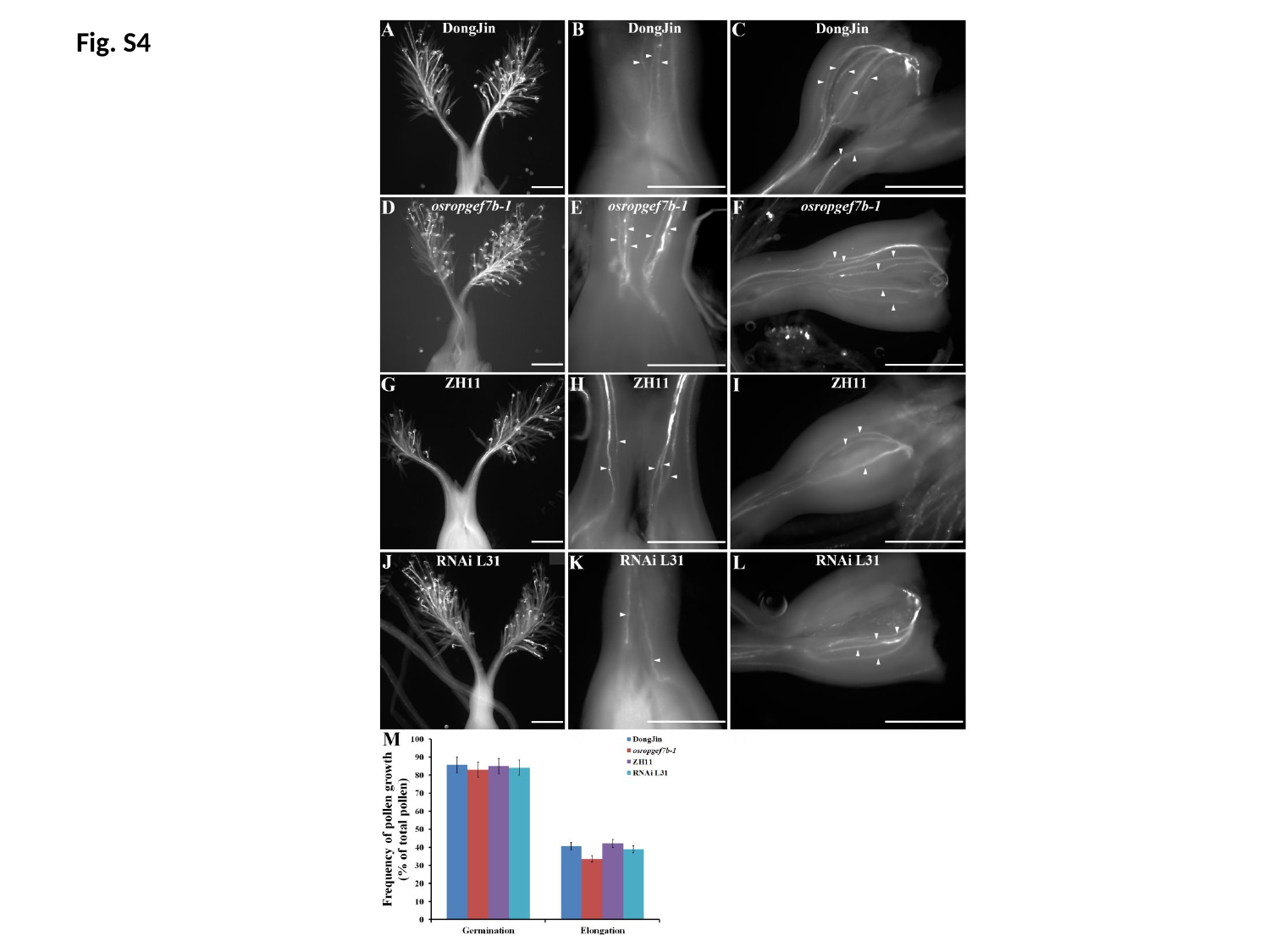

Fig. S4

## Slide 5
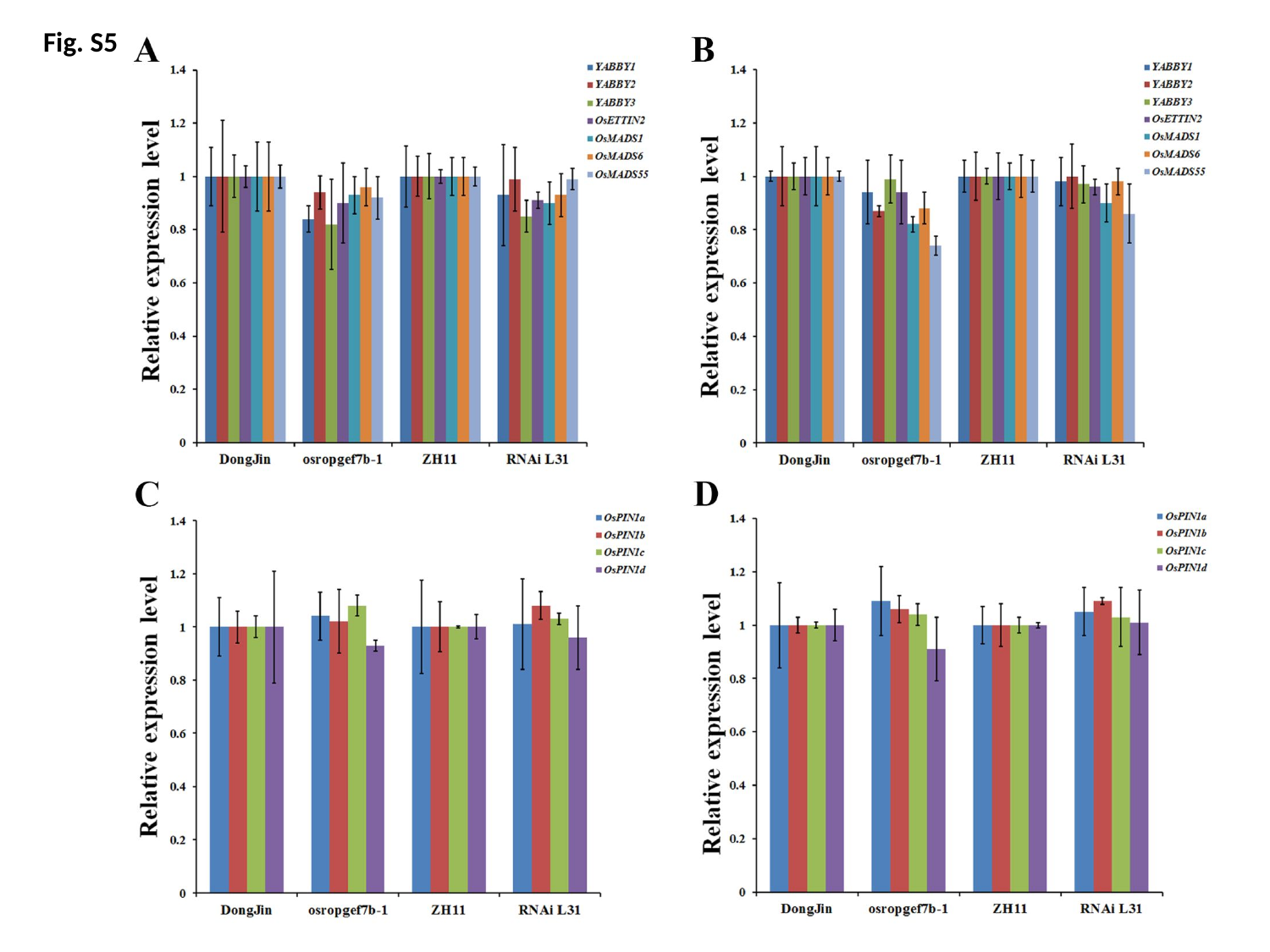

Fig. S5

## Slide 6
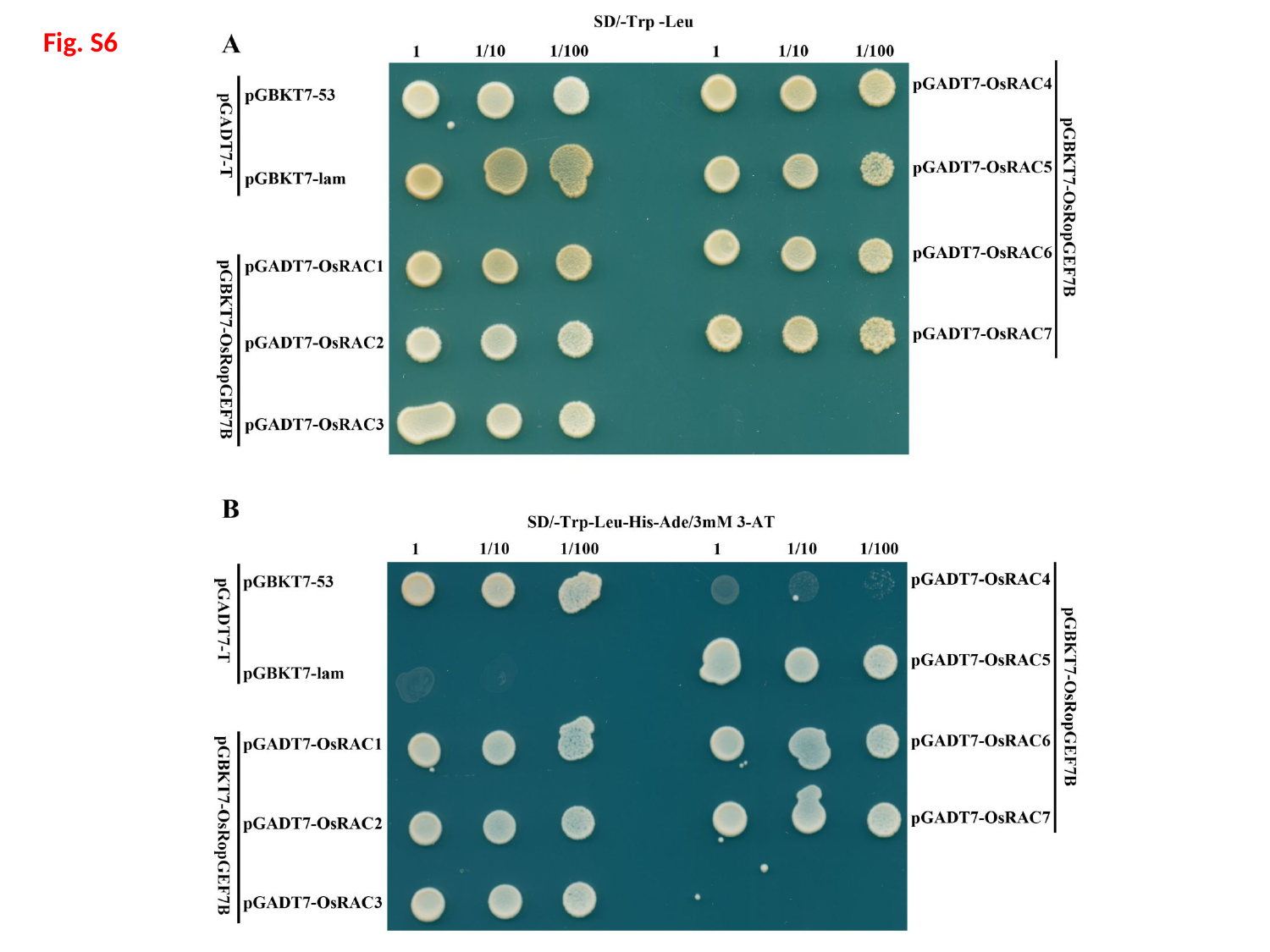

Fig. S6

## Slide 7
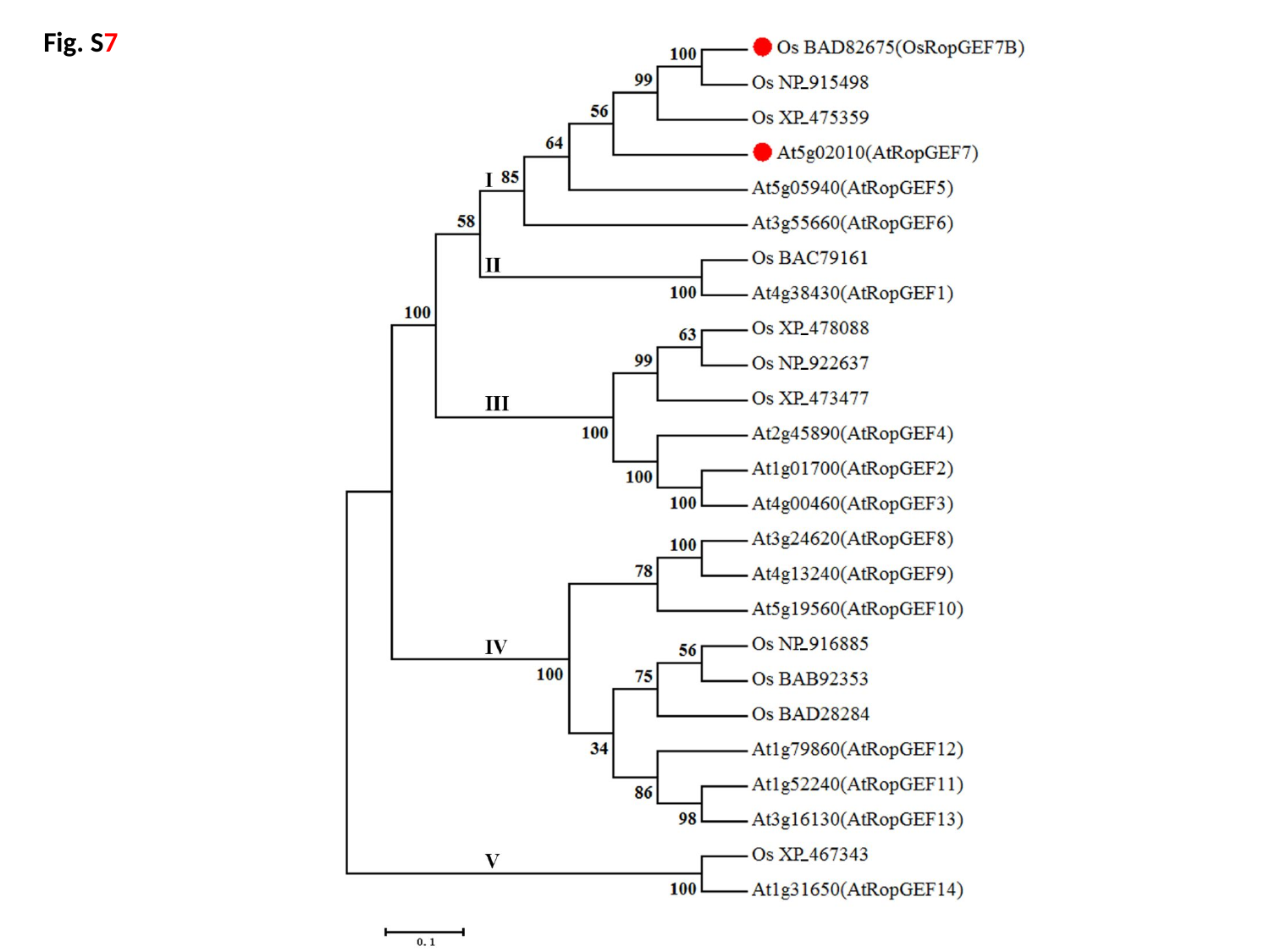

Fig. S7

Supplement: Supplementary file 1 — Figure S1. Relative expression levels of OsRopGEF7B in various tissues of rice at vegetative and reproductive stages. Figure S2. Relative expression levels of OsRopGEF7B in OsRopGEF7B-RNAi lines. Figure S3. OsRopGEF7B does not affect pollen development. Figure S4. In vivo pollen germination and PT elongation. Figure S5. Relative expression levels of a subset of genes associated with floral development in osropgef7b-1 mutant and RNAi L31 line at both seedling and floral stages. Figure S6. Interactions between OsRopGEF7B and OsRACs in the Y2H assay after seven days of growth. Figure S7. Phylogenetic relationships between OsRopGEFs and AtRopGEFs. (PPTX 4833 kb) [file 12284_2018_235_MOESM1_ESM.pptx]
