# Supplementary material for: Guanine Nucleotide Exchange Factor 7B (RopGEF7B) is involved in floral organ development in Oryza sativa
Source: Rice (N Y). 2018 Jul 30;11:42. doi: 10.1186/s12284-018-0235-0 (PMC6066601; doi:10.1186/s12284-018-0235-0)
Supplement: Supplementary file 2 — Table S1. List of primers used in this study. (DOCX 20 kb) [file 12284_2018_235_MOESM2_ESM.docx]

| Experiment | Primer name | Sequence |
| --- | --- | --- |
| *OsRopGEF7B* T-DNA  mutant identification | P1  P2  LB | 5'-TTGAAGCAAAGGTTTCCTGG-3'  5'-TGCACTCCACTATGCTGAGG-3'  5'-CCACAGTTTTCGCGATCCAGACTG-3' |
| Vector construction | *OsRopGEF7B* *GUS*-F  *OsRopGEF7B* *GUS*-R  *OsRopGEF7B* RNAi-F  *OsRopGEF7B* RNAi-R  *OsRopGEF7B* pGADT7-F  *OsRopGEF7B* pGADT7-R  *OsRAC1* PGBKT7-F  *OsRAC1* PGBKT7-R  *OsRAC2* PGBKT7-F  *OsRAC2* PGBKT7-R  *OsRAC3* PGBKT7-F  *OsRAC3* PGBKT7-R  *OsRAC4* PGBKT7-F  *OsRAC4* PGBKT7-R  *OsRAC5* PGBKT7-F  *OsRAC5* PGBKT7-R  *OsRAC6* PGBKT7-F  *OsRAC6* PGBKT7-R  *OsRAC7* PGBKT7-F  *OsRAC7* PGBKT7-R | 5'-TCTTGGACTCCGTACTACAGCTGGC-3'  5'-CGCGTGACGGGGTCTATCTAACTAG-3'  5'-ACATTGTCGCTCGCATCG-3'  5'-CACCGCTGATTATCACCCC-3'  5'-GAATTCATGGGGAGCGGGGAGGAGG-3'  5'-GTCGACTTACAGCTCTGTGGTCGTCGAGACA-3'  5'-GAATTCATGAGCTCGGCGGCGGCG-3'  5'-GGATCCCTACGCGAAACAAGCGCTTCCGC-3'  5'-GAATTCATGAGCGGCGCCACCAAGT-3'  5'-GGATCCCTAAACAACACATGCACTGCCACAC-3'  5'-GAATTCATGGCGTCCAGCGCCTCC-3'  5'-GGATCCTCAGGATTTGAAGCATGACATTTTC-3'  5'-GAATTCATGGCGTCCAGCGCGTC-3'  5'-GGATCCTCACTTGAAGCATACTAGCCTTCTT-3'  5'-GAATTCATGAGCGCGTCTCGGTTCATC-3'  5'-GGATCCTTACAAGATGGCACATCCTTTCTGC-3'  5'-GAATTCATGAGCGCGTCCAGGTTCATAAAGT-3'  5'-GGATCCTCACAAAATGGAGCACGCCCC-3'  5'-GAATTCATGAGCACGGCGAGGTTCATCA-3'  5'-GAGCTCTCATAGGATCCAGCAACTCCTCTGC-3' |
| Real-time PCR | *Actin*-F  *Actin*-R  *OsRopGEF7B*-F  *OsRopGEF7B*-R  *OsMADS1*-F  *OsMADS1*-R  *OsMADS6*-F  *OsMADS6*-R  *OsMADS55*-F  *OsMADS55*-R  *OsETTIN2*-F  *OsETTIN2*-R  *YABBY1*-F  *YABBY1*-R  *YABBY2*-F  *YABBY2*-R  *YABBY3*-F  *YABBY3*-R  *OsPIN1a*-F  *OsPIN1a*-R  *OsPIN1b*-F  *OsPIN1b*-R  *OsPIN1c*-F  *OsPIN1c*-R  *OsPIN1d*-F  *OsPIN1d*-R | 5'-TGTATGCCAGTGGTCGTACCA-3'  5'-CCAGCAAGGTCGAGACGAA-3'  5'-GCTGTTCCACCGTACCAAGT-3'  5'-CCCCCTGTTGCTGTAAGACC-3'  5'-AGGATTTGGGCCCACTAAGC-3'  5'-CCACCATCTTGCCAGGACAT-3'  5'-GATCTTGGACCACTCAGCGT-3'  5'-AGCTTGTGCTTGAGTTGCCT-3'  5'-TCGCTTAATTCGTGCAAGTTATG-3'  5'-AAGTCTCAGCCGAGGTCACAA-3'  5'-TCTCGTCCCTCTACGTGCTT-3'  5'-CCGATTACAACGGGAGCTTA-3'  5'-GTCGGTCCAGTTTACATCGGAGCAT-3'  5'-TGGTGATCTTGAACAGTGCGTGCTT-3'  5'-TGTCAGTGAACTTGCGAGGATTG-3'  5'-TCTCTGTGGCTTATGTCAGGATTGT-3'  5'-AAGGACGAAATCCAACGCATCAA-3'  5'-GCCCTTCCTTTAGTAGCCCATCCTT-3'  5'-AGTACAAAGCTTGGGGGGAC-3'  5'-ATCTCTTGTCAGAATCGGCG-3'  5'-CCCCATCACATTGGTCTACTACAT-3'  5'-TCTTCAATCAGAAACCGTGGC-3'  5'-GCCAAGCAACCAAGCAGCAA-3'  5'-TTCACCACAAGGCCAGCCAAC-3'  5'-CTGAGCACAGCCGTGATATTCG-3'  5'-CTGGCCGTCACTTGAACCC-3' |
